# Highly Divergent *Clostridium difficile* Strains Isolated from the Environment

**DOI:** 10.1371/journal.pone.0167101

**Published:** 2016-11-23

**Authors:** Sandra Janezic, Mojca Potocnik, Valerija Zidaric, Maja Rupnik

**Affiliations:** 1 National Laboratory for Health, Environment and Food (NLZOH), Maribor, Slovenia; 2 University of Maribor, Faculty of Medicine, Maribor, Slovenia; Universidad Andres Bello, CHILE

## Abstract

*Clostridium difficile* is one of the most important human and animal pathogens. However, the bacterium is ubiquitous and can be isolated from various sources. Here we report the prevalence and characterization of *C*. *difficile* in less studied environmental samples, puddle water (n = 104) and soil (n = 79). *C*. *difficile* was detected in 14.4% of puddle water and in 36.7% of soil samples. Environmental strains displayed antimicrobial resistance patterns comparable to already published data of human and animal isolates. A total of 480 isolates were grouped into 34 different PCR ribotypes. More than half of these (52.9%; 18 of 34) were already described in humans or animals. However, 14 PCR ribotypes were new in our PCR ribotype library and all but one were non-toxigenic. The multilocus sequence analysis of these new PCR ribotypes revealed that non-toxigenic environmental isolates are phylogenetically distinct and belong to three highly divergent clades, two of which have not been described before. Our data suggest that environment is a potential reservoir of genetically diverse population of *C*. *difficile*.

## Introduction

*Clostridium difficile* is an important nosocomial pathogen that causes antibiotic-associated diarrhea and pseudomembranous colitis [1,2] and presence of *C*. *difficile* is well documented in hospitalized patients and hospital environment. With increasing number of *C*. *difficile* infections in the community, there is a need to better understand other possible sources for infection. The main natural reservoir of *C*. *difficile* is the gut of young individuals, either humans or animals. But *C*. *difficile* is ubiquitous due to the ability of forming oxygen resistant spores and has been reported from food, water, soil or households [3–14]. Different water sources, such as rivers, sea, lakes, inland drainage, swimming pools, wastewater treatment plants and tap water were positive for *C*. *difficile* [3,8–12]. Soil is less studied environment, but *C*. *difficile* was reported from rural and urban areas [3,9,13,14].

Antimicrobial treatment has a key role in the development of *C*. *difficile* infection. *C*. *difficile* is resistant to a wide range of antimicrobial agents used in daily practice and can colonize the gut in the presence of antimicrobials that disrupts healthy gut microbiota [1]. The resistance to antimicrobials is regularly surveyed for human and animal *C*. *difficile* isolates [15–19] but data on antimicrobial resistance in environmental isolates are sparse. A single publication reported on antimicrobial resistance of *C*. *difficile* strains isolated from estuarine environments [20] but, to the best of our knowledge, there are no publications yet on antimicrobial resistance of isolates from soil and fresh water ecosystems.

The presence of *C*. *difficile* in water and soil may be important if there is an overlap between strains from these environments and strains isolated from symptomatic humans and animals. Different molecular approaches have been used for typing of *C*. *difficile*. Current standard is PCR ribotyping, analysis of variably sized fragments, amplified 16S-23S ribosomal DNA intergenic spacer regions. Another widely used molecular typing method is toxinotyping, a PCR-restriction fragment length polymorphism (PCR-RFLP) based method for differentiating *C*. *difficile* strains according to changes in the PaLoc (pathogenicity locus), a region encoding two main virulence factors, toxin A (TcdA) and toxin B (TcdB) [21]. For identifying phylogenetic relationships and population structure of *C*. *difficile* strains multi locus sequence typing (MLST) is an important tool [22,23].

The aim of this study was to isolate and characterize *C*. *difficile* from two types of environmental samples, soil and puddle water. The genetic and phenotypic diversity of the isolates were assessed using PCR ribotyping, toxinotyping and antibiotic susceptibility testing. In order to better understand evolution of the environmental strains the MLST-based phylogeny was constructed and compared to the population structure of the species.

## Materials and methods

### Sample collection

Puddle water and soil samples were collected in urban and rural areas in eastern parts of Slovenia (S1 Fig). Water samples from puddles were collected between April 2013 and July 2014. In rural areas, samples were collected from large puddles present on fields, meadows, pastures and organic waste pile at local garbage company. Urban samples were collected from puddles on concrete or asphalt terrain or on paving stones, at different locations within a single town. Specific permissions for sampling in rural areas were not required since no national parks or protected areas were included. In urban locations, permission for sampling within the area of large teaching hospital was obtained.

Water was collected into sterile 50 ml centrifuge tubes transferred to the laboratory and stored at 4°C until processing. Altogether, 104 water samples from puddles were collected (44 from rural and 60 from urban locations).

Soil sampling was performed between August 2013 and January 2015. Samples were collected from public areas within a single town and in rural areas from fields, meadows, horse pastures and woods. Surface soil (up to 1 cm deep) was collected with disposable plastic spoons and placed into sterile plastic bags. Samples were transferred to the laboratory and stored at room temperature until processing. Altogether, 79 soil samples were collected (44 from rural and 35 from urban locations).

### Isolation of *C*. *difficile* from puddle water

Pre-treatment of samples and bacterial growth from the filters with heat and ethanol shock, respectively was used to reduce the competing bacteria, to increase the sensitivity of the culture and *C*. *difficile* recovery. Water samples (50 ml) were subjected to a heat shock by incubation at 70°C for 20 min. The entire volume was then filtered through 0.2 μm cellulose nitrate membrane filter (Whatman) using Milipore filtering system. Filters were placed on selective agar chromID® *C*. *difficile* (bioMerieux) and incubated anaerobically at 37°C for 3 days. After incubation, up to 20 presumptive *C*. *difficile* colonies were picked from each filter and subcultured onto blood agar plates (COH, bioMerieux). Remaining bacterial growth was swabbed from the filter, resuspended in 700 μl of absolute ethanol and incubated at room temperature for 30 min. After centrifugation the pellet was inoculated onto chromID® *C*. *difficile* plates and incubated anaerobically for 2 days. Up to 10 colonies with suitable *C*. *difficile* like morphology were subcultured onto COH plates. Isolates were identified using MALDI-TOF (Biotyper, Bruker).

### Isolation of *C*. *difficile* from soil

To maximize recovery of *C*. *difficile*, two slightly different approaches were used, one of which included longer incubation of soil in water (called here soaking), as we expected that water might improve release of *C*. *difficile* spores from soil particles. Therefore, each soil sample was treated in two parallels. Twenty-five grams of soil was resuspended in 90 ml of sterile water in two aliquots; one aliquot was processed immediately, and the other was incubated for one week at room temperature (soaking). Further isolation steps were identical for both treatments (with and without soaking). To remove majority of soil particles, 50 ml of soil suspension was first centrifuged at 50 x g for 2 min. Forty milliliters were transferred to a new sterile tube and again centrifuged at 50 x g for 2 min. Supernatant (30 ml) was subjected to heat shock, at 70°C for 20 min and the entire volume was then filtered through 0.2 μm cellulose nitrate membrane filter. Further isolation procedure was identical as described above for water samples.

### Toxinotyping and PCR ribotyping

Toxinotyping was performed as previously described [24]. Binary toxin gene (*cdt*B) was detected as described by Stubbs *et al*. [25]. The PaLoc-negative genotype was confirmed by PCR using Lok1/Lok3 primers [26]. PCR ribotyping was performed according to the method described previously [27]. PCR ribotypes were determined by comparison of banding patterns with the internal database using the BioNumerics software v7.5 (Applied Maths). Strains that did not match to any of Cardiff/Leeds reference PCR ribotypes represented in our library were designated with an in-house nomenclature (SLO and three-digit code).

### Molecular confirmation of isolates using 16S rDNA sequencing

Genomic DNA used for 16S rDNA amplification and sequencing was extracted using QIAamp DNA Mini Kit (Qiagen, Germany), following manufacturer`s instructions for isolation of Gram positive bacteria. Amplification of the 16S rRNA gene was performed as described previously by Bianciotto *et al*. [28]. Amplified 16S rDNA were sequenced on 3500 Genetic Analyzer using the BigDye Terminator Kit (Applied Biosystems). The forward and reverse strands were aligned using BioNumerics v7.5 (Applied Maths) and the 16S rDNA sequence was then compared with entries in the Ribosomal database Project and 16S rDNA sequences deposited in the GenBank [29,30]. Phylogenetic analyses were conducted in MEGA 6 [31].

### Nucleotide sequence accession numbers

All the 16S rDNA sequences obtained have been submitted to the GenBank with accession numbers KX792123 to KX792138.

### MLST analysis

Seven housekeeping genes were extracted from *C*. *difficile* genomes (MiSeq, Illumina) and the allelic numbers and MLST sequence types (MLST STs) were assigned using the PubMLST *C*. *difficile* database. New alleles were submitted to the PubMLST database (http://pubmlst.org/cdifficile/) after which allele numbers and new STs were assigned. Additional 29 STs, representing the *C*. *difficile* population, were downloaded from the PubMLST database. Concatenated sequences were aligned by Clustal Omega (http://www.ebi.ac.uk/Tools) and maximum likelihood tree was constructed using MEGA version 6 [31].

### Antimicrobial susceptibility testing

Antimicrobial susceptibility testing was performed by broth microdilution using custom designed 96-well Micronaut-S CD MIC plates (Merlin Diagnostics), following manufacturer’s recommendations. Fifteen antimicrobials were tested (imipenem, erythromycin, daptomycin, clindamycin, tetracycline, rifampicin, tigecycline, moxifloxacin, metronidazole, vancomycin, fusidic acid, amoxicillin, linezolid, ceftriaxone and levofloxacin). The epidemiological cut-off values (ECOFF) for reduced susceptibility were defined according to European Committee on Antimicrobial Susceptibility Testing (EUCAST) [32]. If ECOFF values were not available, clinical breakpoints according to Clinical and Laboratory Standards Institute (CLSI) (M100S, 2016) recommendations were used [33].

## Results

### Detection and characterization of *C*. *difficile*

The overall isolation rate of *C*. *difficile* in environmental samples was 24.0% (44 positive samples of 183 samples tested). *C*. *difficile* was isolated from 15 (14.4%) of 104 puddle water samples and from 29 (36.7%) of 79 soil samples.

Altogether 480 isolates were recovered (361 from soil and 119 from puddles) and distributed into 34 distinct PCR ribotypes (Table 1). Of these, only 12 (35.3%) could be assigned to one of the internationally recognized ribotypes. The remaining 22 profiles could not be assigned PCR ribotype based on our library having 71 Cardiff/Leeds reference strains and were given an in-house designation. Fourteen of detected PCR ribotypes did not match with any PCR ribotype in our collection so far isolated from humans, animals or the environment.

**Table 1:** Overview of *C*. *difficile* genotypes isolated from soil and puddles with number of isolates obtained and number of sampling sites where the genotype was found.

PCR ribotype	Toxinotype^1^	Puddle water^2^	Soil^2^	PCR ribotype found in	
				humans	animals
002	0	7/1		+	+
005	0		10/1	+	+
010	Tox-, lok 1/3+	12/1	80/6	+	+
012	0		1/1	+	+
014/020	0	26/3	81/7	+	+
015	Tox-, lok 1/3+	11/1		+	+
023	IV (CDT+)	2/1	8/1	+	+
029	0		9/1	+	+
103	0		20/2	+	+
153(CE)	XId (CDT+)	5/1		-	-
244	IXb (CDT+)	1/1		+	+
251	IIIb (CDT+)	9/1	5/1	+	+
SLO 002	Tox-, lok 1/3+	10/1		+	+
SLO 029	0		5/1	+	+
SLO 069	0	12/2		+	+
SLO 092	Tox-, lok 1/3+		2/1	-	-
SLO 187	XId (CDT+)	9/1		+	-
SLO 191	0	5/1		+	-
SLO 192	Tox-, lok 1/3+	7/3		+	-
SLO 204	Tox-, lok 1/3-	2/1	16/5	-	-
SLO 205	Tox-, lok 1/3-		19/3	-	-
SLO 206	Tox-, lok 1/3-		9/1	-	-
SLO 208	Tox-, lok 1/3-		1/1	-	-
SLO 214	Tox-, lok 1/3-		11/1	-	-
SLO 215	Tox-, lok 1/3-		4/1	-	-
SLO 216	Tox-, lok 1/3-		8/1	-	-
SLO 218	Tox-, lok 1/3-		13/1	-	-
SLO 221	Tox-, lok 1/3-	1/1		-	-
SLO 222	Tox-, lok 1/3-		10/1	-	-
SLO 223	Tox-, lok 1/3-		12/2	-	-
SLO 229	Tox-, lok 1/3-		12/2	+	-
SLO 230	XXXII		15/1	-	-
SLO 240	Tox-, lok 1/3-		9/1	-	-
SLO 251	Tox-, lok 1/3-		1/1	-	-
Total nr. of isolates/total nr. of *C*. *difficile* positive samples		119/15	361/29	na	na
Nr. of PCR ribotypes		15	24	na	na
Nr. of tested samples		104	79	na	na

^1^Tox—refers to PaLoc negative strains; lok1/3+ refers to strains where 115-bp sequence, replacing the PaLoc was PCR amplified; lok1/3—refers to PaLoc negative strains where 115-bp sequence could not be amplified with primers lok1/lok3; CDT+ indicates presence of binary toxin genes

^2^number of isolates/number of sampling sites; na—not applicable

In 12 out of 44 positive samples, multiple PCR ribotypes were detected; four of these were from puddles and eight from soil. Up to four different PCR ribotypes were isolated from a single soil sample and in puddle water up to three different PCR ribotypes could be detected in a single sample.

Overall, the three most common PCR ribotypes were 014/020, 010 and SLO 204 which were found in 10, 7 and 6 samples from puddles and/or soil, respectively. More than half of PCR ribotypes (18 of 34; 52.9%) that were found in the environment were previously described in humans and of these 14 were also found in animals (Table 1). Sixteen PCR ribotypes from soil and puddles had no matching profile from humans or animals in our collection. The majority of these ribotypes was isolated from soil samples only (14 out of 16) (Table 1).

### Detection of toxin genes and toxinotyping

Among the 34 PCR ribotypes identified, 19 were non-toxigenic (PaLoc-negative) and 15 PCR ribotypes included toxigenic isolates.

Large proportion of non-toxigenic strains belonged to PCR ribotypes which were newly found in this study in soil. A 115-bp sequence, that is normally found replacing the PaLoc in non-toxigenic strains, could not be PCR amplified in 14 of 19 PaLoc negative ribotypes (Table 1). The lack of amplification was most likely due to insertions other than PaLoc described recently in some clinical isolates [34–36].

Toxigenic isolates belonged to six different toxinotypes 0, IIIb, IV, IXb, XId and XXXII; two of them (IXb and XId) are here newly described (see below). Although six different toxinotypes were identified, more than half (9 out of 15) of toxigenic PCR ribotypes belonged to toxinotype 0. Four of the six toxinotypes were positive for binary toxin gene (Table 1).

Two new variant toxinotypes were identified in this study, IXb and XId, both binary toxin CDT positive. Both were already included in the updated toxinotyping scheme [37], but are here described more detailed. Toxinotype IXb (PCR ribotype 244) is similar to the reference strain of toxinotype IX in the main fragments B1 and A3 (most variable regions in *tcd*B and *tcd*A, coding for catalytic and binding domain, respectively). Further distribution into subtypes, designated from IXa to IXd, is based on *Hind*III in *Rsa*I RFLP of B2 region of *tcd*B gene. Another new toxinotype, XId (PCR ribotype 153(CE)), differed from other toxinotype XI strains (XIa-c) in different RFLP pattern of A3 fragment of *tcdA* gene. A truncated PaLoc, with just a part of 3’ end present (equivalent to A3 fragment), characterizes toxinotype XI strains, corresponding to A-B-CDT+ phenotype [37].

### Antimicrobial susceptibility

Environmental strains showed a range of antimicrobial susceptibility to different antibiotics. Resistance to imipenem (37.1% of isolates), erythromycin (8.6%) and clindamycin (28.6%) and reduced susceptibility (ECOFF according to EUCAST) for tetracycline (8.6%), rifampicin (8.6%) and daptomycin (14.3%) was observed (Table 2). Reduced susceptibility to tetracycline and rifampicin was observed only in non-toxigenic isolates. Combined reduced susceptibility and/or resistance to three antibiotics was found in four strains, belonging to PCR ribotypes SLO 002 (PaLoc neg.), SLO 192 (PaLoc neg.), 244 (IXb) and 251 (IIIb).

**Table 2:** Antibiotic resistance and/or reduced susceptibilities of environmental *C*. *difficile* isolated against 15 antimicrobial agents.

Antibiotic	Clinical breakpoint (mg/L)^a^	ECOFF (mg/L)^b^	Isolates with reduced susceptibility (n = 35) ^b^	Resistant isolates (n = 35) ^a^	PCR ribotypes
Imipenem	≥16	nd	na	13 (37.1%)	005, 023, 244, 251, SLO 029, SLO 069, SLO 191, SLO 192, SLO 204, SLO 205, SLO 214, SLO 218
Erythromycin	≥8	>2	12 (34.3%)	3 (8.6%)	005, 014/020, 015, 103, 244, 251, SLO 002, SLO 191, SLO 192, SLO 214, SLO 218, SLO 222
Daptomycin	nd	>4	5 (14.3%)	na	005, 244, 251, SLO 218, SLO 229
Clindamycin	≥8	>16	3 (8.6%)	10 (28.6%)	103, SLO 002, SLO 222
Tetracycline	≥16	>0.25	3 (8.6%)	0.0	SLO 002, SLO 192, SLO 240
Rifampicin	≥4	>0.004	3 (8.6%)	0.0	SLO 229, SLO 240
Tigecycline	ND	>0.25	0.0	na	na
Moxifloxacin	≥8	>4	0.0	0.0	na
Metronidazole	≥32	>2	0.0	0.0	na
Vancomycin	≥16	>2	0.0	0.0	na
Fusidic acid	nd	>2	0.0	na	na
Amoxicillin	≥16	nd	0.0	0.0	na
Linezolid	≥8	nd	0.0	0.0	na
Ceftriaxone	≥64	nd	0.0	0.0	na
Levofloxacin	≥8	nd	0.0	0.0	na

^a^based on Clinical and Laboratory Standards Institute (CLSI)

^b^ECOFF—Epidemiological cut-off values according to EUCAST; na—not applicable

### Multilocus sequence analysis

**Fig 1:**
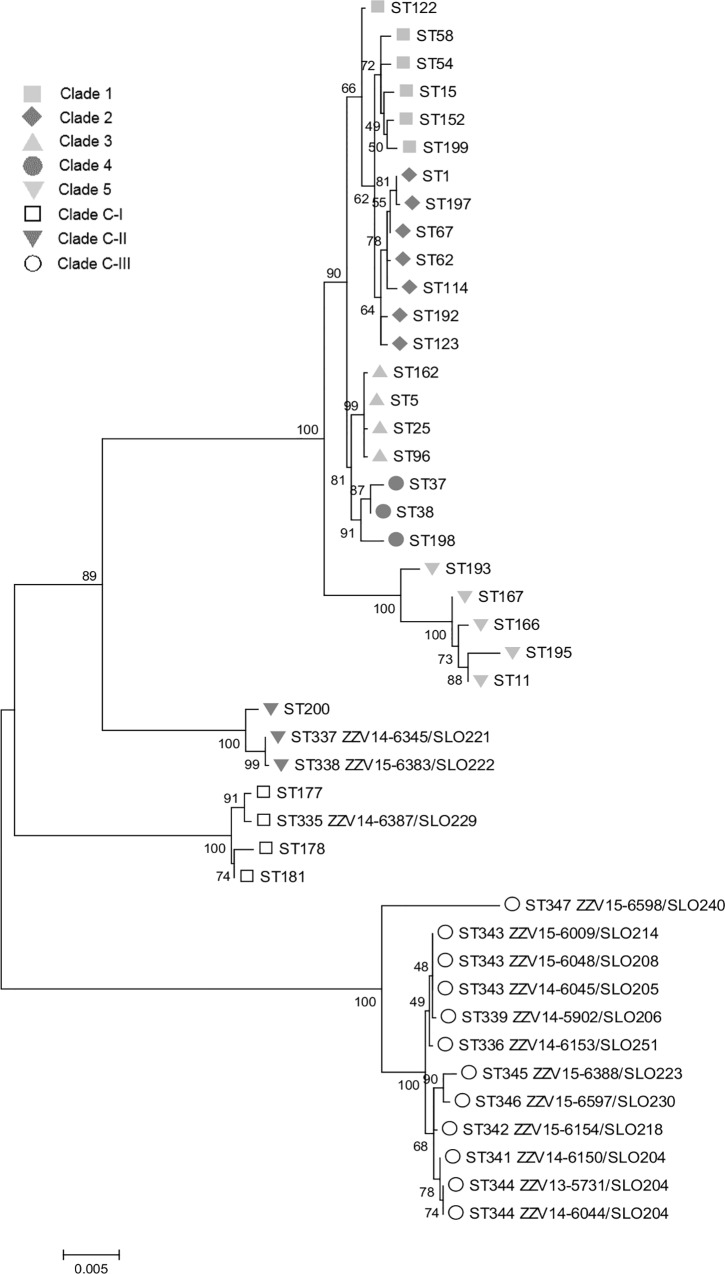
Phylogenetic relationship of *C*. *difficile* based on MLST sequences. The Maximum likelihood phylogenetic tree was constructed based on the alignment of concatenated DNA sequences of the seven housekeeping genes. Clades 1–5 and C-I were already described and clades C-II and C-III are new. In clade C-II a strain with ST200 (toxinotype XXXII) is present and is described in our recent publication [36].

Representatives of PCR ribotypes which were newly identified in this study were further analyzed by MLST. Identity of these isolates was confirmed by the 16S rDNA analysis (S1 Table) and analysis of two additional genes *rpo*B (S2 Table, S2 Fig) and *gyr*B (S3 Table and S3 Fig). Fifteen strains, one toxigenic and 14 non-toxigenic (where 115-bp insertion could not be PCR amplified) belonging to 13 distinct ribotypes (for PCR ribotype SLO 204, three isolates were included) were sequenced and their MLST- sequence types were determined from the sequences. Twelve sequence types were identified, all of which were new (ST 335 to ST339 and ST341 to ST347, Table 3). Phylogenetic tree based on concatenated MLST sequences of environmental strains and representatives of all previously described clades [34] demonstrated two new, highly divergent lineages, here designated as C-II and C-III (following the designations introduced by Dingle *et al*. [34]). Clade C-II included two isolates and clade C-III contained 12 isolates represented by 10 different PCR ribotypes, all but one (toxinotype XXXII (A-B+), PCR ribotype SLO 240) were non-toxigenic. Only a single strain was found in recently described clade C-I (Fig 1).

**Table 3:** Multilocus sequence types and allelic profiles of environmental isolates.

PCR ribotype	Isolate	ST	Clade	MLST allelic profile						
				*adk*	*atpA*	*dxr*	*glyA*	*recA*	*sodA*	*tpi*
SLO 204	ZZV14-6150	341	C-III	26	35	39	52	31	49	52
SLO 204	ZZV13-5731	344	C-III	26	36	39	52	31	49	52
SLO 204	ZZV14-6044	344	C-III	26	36	39	52	31	49	52
SLO 205	ZZV14-6045	343	C-III	26	36	37	54	31	49	52
SLO 206	ZZV14-5902	339	C-III	26	35	37	54	31	49	52
SLO 208	ZZV15-6048	343	C-III	26	36	37	54	31	49	52
SLO 214	ZZV15-6009	343	C-III	26	36	37	54	31	49	52
SLO 218	ZZV15-6154	342	C-III	26	35	39	54	31	47	50
SLO 221	ZZV14-6345	337	C-II	25	21	38	53	21	36	53
SLO 222	ZZV15-6383	338	C-II	25	21	38	53	21	48	53
SLO 223	ZZV15-6388	345	C-III	27	37	39	55	31	49	54
SLO 229	ZZV14-6387	335	C-I	13	17	22	33	18	31	51
SLO 230	ZZV15-6597	346	C-III	27	38	40	56	31	49	55
SLO 240	ZZV15-6598	347	C-III	28	39	41	57	30	50	56
SLO 251	ZZV14-6153	336	C-III	24	36	37	54	31	49	50

## Discussion

The ubiquity of *C*. *difficile* is well known, but the studies describing its presence in water and soil are not numerous, and only a few of them also include molecular characterization of strains and antibiotic resistances. The purpose of this study was therefore to determine occurrence and variability of *C*. *difficile* genotypes isolated from soil and water from puddles.

Our results with 14.4% of positive puddle water samples and 36.7% positive soil samples are in agreement with previously reported *C*. *difficile* isolation rates from soil (1 to 37%) [3,9,13,14] and from various water ecosystems (lakes, rivers, swimming pools, tap water, waste water treatment plants) (27% to 100%) [3,7–11]. The highest percent of water positivity was found in waste water treatment plants, where all samples were positive on *C*. *difficile* in two different studies [11, 12].

To the best of our knowledge, this is the first report presenting data of antimicrobial susceptibility patterns in soil and water isolates. In our study resistance or reduced susceptibilities to imipenem, erythromycin, clindamycin, tetracycline, rifampicin and daptomycin were observed, which is comparable to already published data of human and animal isolates [16,18,19]. In this study, none of the environmental strains was resistant to fluoroquinolones, as is known for some epidemic strains circulation in human population [17].

Multiple resistance was rare and was found in only four strains, three of which (PCR ribotypes 251, 244 and SLO 002,) are associated with human and animals hosts (Table 1).

The overlap of *C*. *difficile* PCR ribotypes isolated from humans and animals and from soil and water reported previously, and in this study, indicates exchange between humans, animals and the environment. Transmission could include exposure to animals, fertilizing, irrigation with recycled water, airborne dissemination of spores, or introduction of bacteria to domestic environment by vegetables.

On the other hand, we report here for the first time that a large part of *C*. *difficile* population isolated from soil samples is unique. Fourteen of 24 PCR ribotypes isolated from soil were new in our strain collection, which includes > 5000 *C*. *difficile* isolates (250 different PCR ribotypes) from humans, animals and the environment. Additionally, most of these new PCR ribotypes were non-toxigenic and also differ in their chromosomal PaLoc insertion region from non-toxigenic strains isolated from humans and animals. Human and animal strains characteristically have a short 115-bp insertion, replacing the PaLoc, which can be amplified with specific PCR. In the majority of non-toxigenic soil strains amplification of the 115-bp insertion was not successful, most likely due to larger insertions (not further characterized). Sporadic strains with such characteristic were already reported from human cases [34–36].

To assess the placement of these new PCR ribotypes within the *C*. *difficile* population a MLST-based phylogeny was performed which demonstrated that strains isolated from soil belonging to new PCR ribotypes (and new MLST sequence types) occupy three distinct, highly divergent clades of *C*. *difficile* population (Fig 1). One of these clades (clade C-I), was already described [34]. Initially, it was associated primarily with non-toxigenic strains but in recent publication also toxigenic strains were found within this clade [38]. For clade C-II a single isolate was so far reported [36], while clade C-III was not described previously. The detailed analysis of 16S rDNA and some other phylogetically relevant genes within *C*. *difficile* and comparison with representatives of some closely related species confirms that isolates from these new clades are highly divergent but could still be identified as *C*. *difficile*. The high abundance of isolates from these divergent clades (C-I to C-III) in the environmental samples and only sporadic isolation from clinical samples indicate that these strains could represent native environmental isolates, which are not primarily associated with humans or animals.

In summary, our results suggest that variability of *C*. *difficile* in puddle water and in soil is higher than known so far. Some soil and water associated *C*. *difficile* strains overlap well with human and animal reservoir however, part of the population in soil is characterized by prevalence of non-toxigenic, highly divergent strains that could represent native environmental strains that have not yet been introduced to human or animal population.

## Supporting Information

S1 Fig Locations of sampling sites of soil and puddle water. Sampling sites are marked with grey circles and one large grey area which indicates the location of several sampling sites (n = 139).
(PDF)

S2 Fig Phylogenetic relationship of *C*. *difficile* strains based on *rpo*B gene sequences. The maximum likelihood phylogenetic tree was constructed in MEGA 6.
(PDF)

S3 Fig Phylogenetic relationship of *C*. *difficile* strains based on *gyr*B gene sequences. The maximum likelihood phylogenetic tree was constructed in MEGA 6.
(PDF)

S1 Table 16S rDNA sequence similarities of the non-toxigenic environmental *C*. *difficile* isolates with type strains of *C*. *difficile* and other closely related bacteria. (PDF)

S2 Table Comparisons of *rpo*B gene similarities in strains investigated. (PDF)

S3 Table Comparisons of *gyr*B gene similarities in strains investigated. (PDF)
