# Supplementary material for: Highly Divergent Clostridium difficile Strains Isolated from the Environment
Source: PLoS One. 2016 Nov 23;11(11):e0167101. doi: 10.1371/journal.pone.0167101 (PMC5120845; doi:10.1371/journal.pone.0167101)
Supplement: S1 Fig — Sampling sites are marked with grey circles and one large grey area which indicates the location of several sampling sites (n = 139). (PDF) [file pone.0167101.s001.pdf]

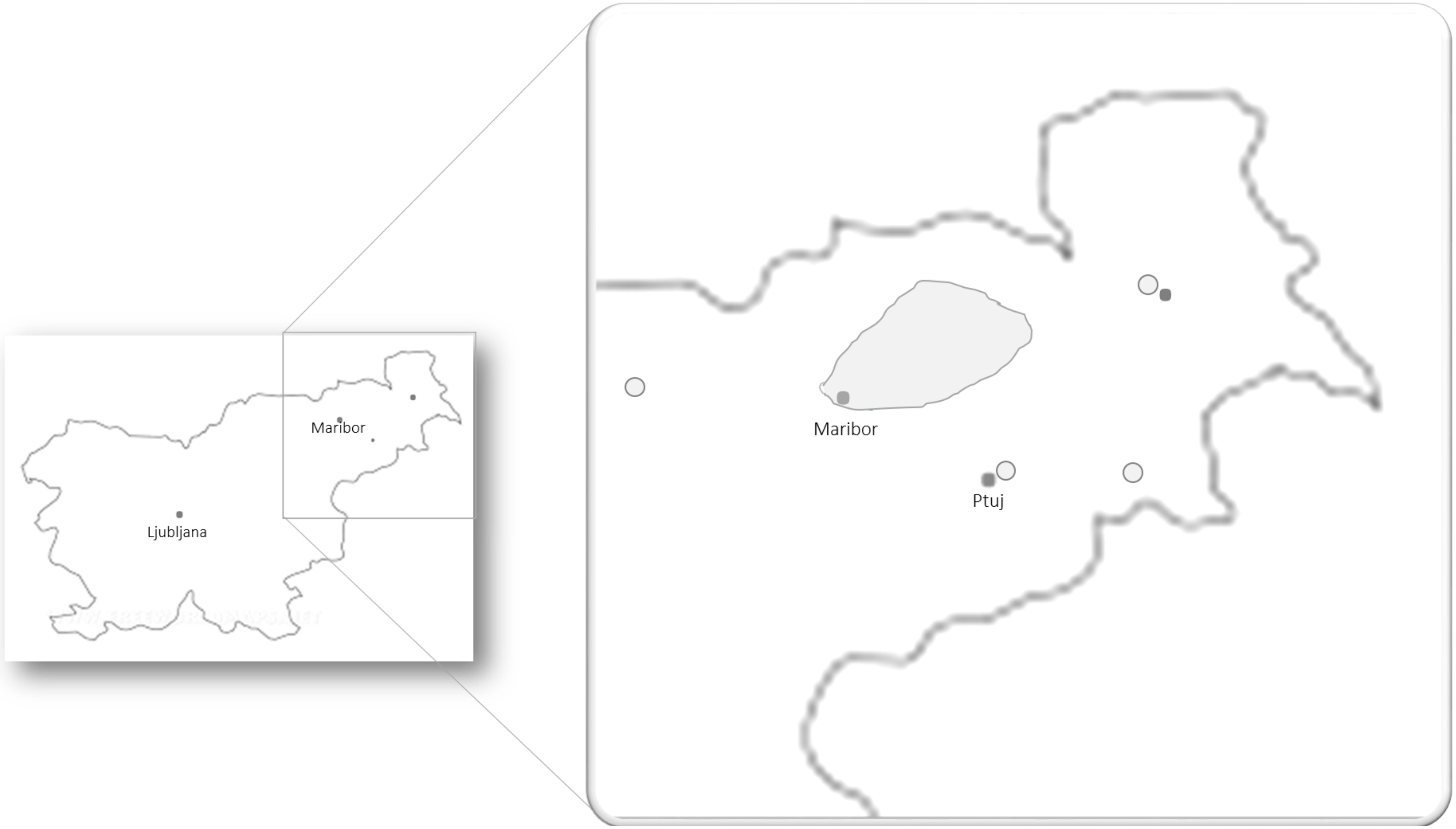

Figure S1. Locations of sampling sites of soil and puddle water. Sampling sites are marked with grey circles and one large grey area which indicates the location of several sampling sites (n=139).
