# Supplementary material for: Highly Divergent Clostridium difficile Strains Isolated from the Environment
Source: PLoS One. 2016 Nov 23;11(11):e0167101. doi: 10.1371/journal.pone.0167101 (PMC5120845; doi:10.1371/journal.pone.0167101)
Supplement: S2 Fig — The maximum likelihood phylogenetic tree was constructed in MEGA 6. (PDF) [file pone.0167101.s002.pdf]

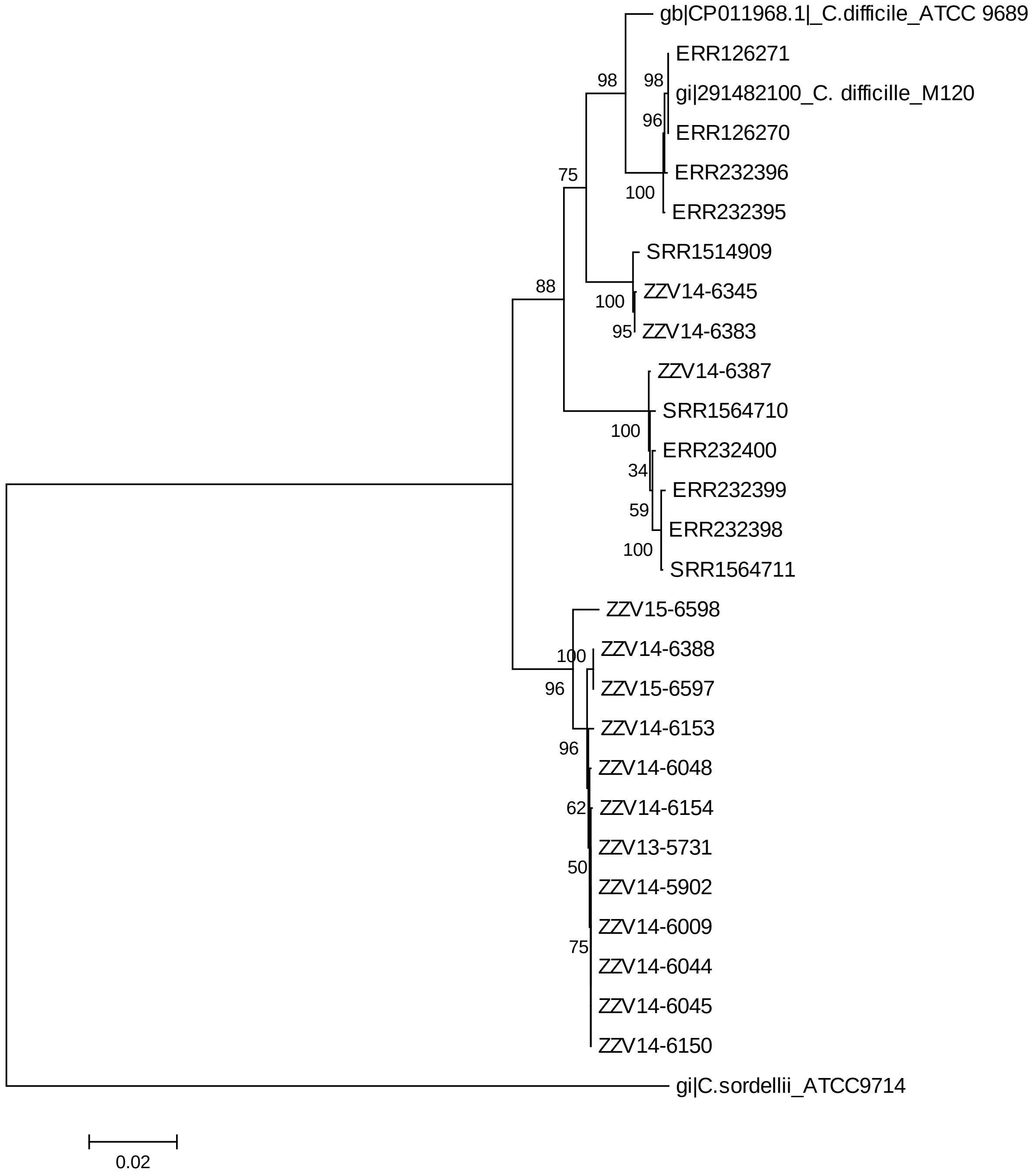

Figure S2. Phylogenetic relationship of *C. difficile* strains based on *rpo*B gene sequences. The maximum likelihood phylogenetic tree was constructed in MEGA 6.
