# Supplementary material for: Highly Divergent Clostridium difficile Strains Isolated from the Environment
Source: PLoS One. 2016 Nov 23;11(11):e0167101. doi: 10.1371/journal.pone.0167101 (PMC5120845; doi:10.1371/journal.pone.0167101)
Supplement: S1 Table — (PDF) [file pone.0167101.s004.pdf]

Table S1. 16S rDNA sequence similarities of the non-toxigenic environmental *C. difficile* isolates with type strain of *C. difficile* and other closely related bacteria.

Isolate	Clade	16S rDNA Sequence length (bp)	*Clostridium difficile,* ATCC 9689[1]	*Eubacterium tenue,* ATCC 25553[2]	*Clostridium sordellii,* ATCC 9714[3]	*Clostridium ghonii,* JCM 1400[4]	*Clostridium mangenotii,* DSM1289[5]	*Peptostreptococcus anaerobius,* NCTC 11460[6]	*Clostridium hiranonis,* TO-931[7]
ZZV14-6387	C-I	1355	99.9	95.9	96.4	95.9	95.1	92.1	94.5
ZZV14-6345	C-II	1344	99.7	96.1	96.4	95.9	95.3	92.1	94.5
ZZV14-6383	C-II	1360	99.6	96.1	96.4	95.9	95.3	92.1	94.5
ZZV14-5902	C-III	1353	99.7	95.9	96.3	95.8	95.2	91.9	94.4
ZZV15-6597	C-III	1356	99.8	95.9	96.3	95.8	95.2	91.9	94.4
ZZV15-6598	C-III	1356	99.8	95.9	96.3	95.8	95.2	92.0	94.4
ZZV14-6009	C-III	1364	99.9	95.9	96.3	95.8	95.2	91.9	94.4
ZZV14-6044	C-III	1355	99.9	95.9	96.3	95.8	95.2	91.9	94.4
ZZV14-6153	C-III	1338	99.9	95.9	96.3	95.8	95.2	91.9	94.4
ZZV14-6154	C-III	1352	99.9	95.9	96.3	95.8	95.2	91.9	94.4
ZZV13-5731	C-III	1346	99.8	95.9	96.3	95.8	95.2	91.9	94.4
ZZV14-6045	C-III	1348	99.9	95.9	96.3	95.8	95.2	91.9	94.4
ZZV14-6048	C-III	1349	99.9	95.9	96.3	95.8	95.2	91.9	94.4
ZZV14-6150	C-III	1345	99.9	95.9	96.3	95.8	95.2	91.9	94.4
ZZV14-6388	C-III	1345	99.9	95.9	96.3	95.8	95.2	91.9	94.4

[1]Accession number: CP011968.1

[2]Accession number: M59118.1

[3]Accession number: AB075771.1

[4]Accession number: AB542933.1

[5]Accession number: FR733662.1

[6]Accession number: AY326462.1

[7]Accession number: AB023970.1
