# Supplementary material for: Highly Divergent Clostridium difficile Strains Isolated from the Environment
Source: PLoS One. 2016 Nov 23;11(11):e0167101. doi: 10.1371/journal.pone.0167101 (PMC5120845; doi:10.1371/journal.pone.0167101)
Supplement: S2 Table — (PDF) [file pone.0167101.s005.pdf]

**Table S2. Comparisons of *rpo*B gene similarities in strains investigated.**

| | | *rpo*B similarity (%) | | |
|---|---|---|---|---|
| **Strain** | **Clade** | **ATCC 9689[3]** | **M120[4]** | **ATCC 9714[5]** |
| ATCC 9689 (T)[3] | 1 | - | 98,5 | 84,9 |
| ERR126270[2] | 5 | 98.5 | 100.0 | 84.5 |
| ERR126271[2] | 5 | 98.5 | 100.0 | 84.5 |
| ERR232395[2] | 5 | 98.6 | 99.9 | 84.5 |
| ERR232396[2] | 5 | 98.5 | 99.9 | 84.5 |
| M120[4] | 5 | 98.5 | - | 84.5 |
| ERR232398[2] | C-I | 96.6 | 96.3 | 84.4 |
| ERR232399[2] | C-I | 96.6 | 96.2 | 84.4 |
| ERR232400[2] | C-I | 96.6 | 96.3 | 84.3 |
| ZZV14-6387 | C-I | 96.7 | 96.4 | 84.3 |
| SRR1564710[1] | C-I | 96.7 | 96.4 | 84.3 |
| SRR1564711[1] | C-I | 96.6 | 96.3 | 84.4 |
| ZZV14-6345 | C-II | 97.5 | 97.2 | 84.2 |
| ZZV14-6383 | C-II | 97.5 | 97.2 | 84.2 |
| SRR1514909[1] | C-II | 97.4 | 97.2 | 84.3 |
| ZZV13-5731 | C-III | 95.8 | 95.4 | 84.9 |
| ZZV14-5902 | C-III | 95.8 | 95.4 | 84.9 |
| ZZV14-6009 | C-III | 95.8 | 95.4 | 84.9 |
| ZZV14-6044 | C-III | 95.8 | 95.4 | 84.9 |
| ZZV14-6045 | C-III | 95.8 | 95.4 | 84.9 |
| ZZV14-6048 | C-III | 95.8 | 95.5 | 84.9 |
| ZZV14-6150 | C-III | 95.8 | 95.4 | 84.9 |
| ZZV14-6153 | C-III | 95.7 | 95.3 | 84.7 |
| ZZV14-6154 | C-III | 95.7 | 95.4 | 84.9 |
| ZZV14-6388 | C-III | 95.8 | 95.5 | 84.9 |
| ZZV15-6597 | C-III | 95.8 | 95.5 | 84.9 |
| ZZV15-6598 | C-III | 95.8 | 95.5 | 84.7 |

[1] Strains described in Monot *et al*. 2015, Sci Rep; [2] strains described in Dingle *et al*., 2014 Genome Biol Evol.

[3] Accession nr. of *C. difficile* ATTC 9689 (type strain): CP011968.1

[4] Accession nr. of *C. difficile* strain M120: FN665653.1

[5] Accession nr. of *C. sordellii* strain ATCC 9714: LN679998.1
