# Supplementary material for: Highly Divergent Clostridium difficile Strains Isolated from the Environment
Source: PLoS One. 2016 Nov 23;11(11):e0167101. doi: 10.1371/journal.pone.0167101 (PMC5120845; doi:10.1371/journal.pone.0167101)
Supplement: S3 Table — (PDF) [file pone.0167101.s006.pdf]

Table S3. Comparisons of *gyr*B gene similarities in strains investigated.

		***gyr*B similarity (%)**		
	**Clade**	**ATCC 9689(T)[3]**	**M120[4]**	**ATCC 9714[5]**
ATCC 9669(T)[3]	1	-	97.3	78.3
ERR126270[2]	5	97.3	100.0	78.2
ERR126271[2]	5	97.3	100.0	78.2
ERR232395[2]	5	97.4	99.9	78.2
ERR232396[2]	5	97.4	99.9	78.3
M120[4]	5	97.3	-	78.2
ERR232398[2]	C-I	94.9	94.3	78.2
ERR232399[2]	C-I	95.1	94.5	78.2
ERR232400[2]	C-I	94.9	94.3	78.2
ZZV14-6387	C-I	94.9	94.3	78.2
SRR1564710[1]	C-I	94.7	94.2	78.3
SRR1564711[1]	C-I	94.9	94.3	78.2
ZZV14-6345	C-II	94.9	93.9	79.0
ZZV14-6383	C-II	94.9	93.9	79.0
SRR1514909[1]	C-II	95.0	93.9	79.2
ZZV13-5731	C-III	93.0	92.6	79.6
ZZV14-5902	C-III	93.0	92.6	79.6
ZZV14-6009	C-III	93.0	92.6	79.6
ZZV14-6044	C-III	93.0	92.6	79.6
ZZV14-6045	C-III	93.0	92.6	79.6
ZZV14-6048	C-III	93.0	92.6	79.6
ZZV14-6150	C-III	93.0	92.6	79.6
ZZV14-6153	C-III	93.0	92.5	79.6
ZZV14-6154	C-III	93.0	92.6	79.6
ZZV14-6388	C-III	93.4	93.0	79.7
ZZV15-6597	C-III	93.4	93.0	79.7
ZZV15-6598	C-III	93.0	92.5	79.4

[1] Strains described in Monot *et al*. 2015, Sci Rep; [2] strains described in Dingle *et al*., 2014 Genome Biol Evol.

[3] Accession nr. of *C. difficile* ATTC 9689 (type strain): CP011968.1

[4] Accession nr. of *C. difficile* strain M120: FN665653.1

[5] Accession nr. of *C. sordellii* strain ATCC 9714: LN679998.1
